# Valpromide Inhibits Lytic Cycle Reactivation of Epstein-Barr Virus

**DOI:** 10.1128/mBio.00113-16

**Published:** 2016-03-01

**Authors:** Kelly L. Gorres, Derek Daigle, Sudharshan Mohanram, Grace E. McInerney, Danielle E. Lyons, George Miller

**Affiliations:** a Department of Molecular Biophysics and Biochemistry, Yale University School of Medicine, New Haven, Connecticut, USA; b Department of Microbiology, Yale University School of Medicine, New Haven, Connecticut, USA; c Department of Pediatrics, Yale University School of Medicine, New Haven, Connecticut, USA; d Department of Epidemiology and Public Health, Yale University School of Medicine, New Haven, Connecticut, USA

## Abstract

Reactivation of Epstein-Barr virus (EBV) from latency into the lytic phase of its life cycle allows the virus to spread among cells and between hosts. Valproic acid (VPA) inhibits initiation of the lytic cycle in EBV-infected B lymphoma cells. While VPA blocks viral lytic gene expression, it induces expression of many cellular genes, because it is a histone deacetylase (HDAC) inhibitor. Here we show, using derivatives of VPA, that blockade of EBV reactivation is separable from HDAC inhibition. Valpromide (VPM), an amide derivative of valproic acid that is not an HDAC inhibitor, prevented expression of two EBV genes, BZLF1 and BRLF1, that mediate lytic reactivation. VPM also inhibited expression of a viral late gene, but not early genes, when BZLF1 was exogenously expressed. Unlike VPA, VPM did not activate lytic expression of Kaposi’s sarcoma-associated herpesvirus. Expression of cellular immediate-early genes, such as FOS and EGR1, is kinetically upstream of the EBV lytic cycle. VPM did not activate expression of these cellular immediate-early genes but decreased their level of expression when induced by butyrate, an HDAC inhibitor. VPM did not alter expression of several other cellular immediate-early genes, including STAT3, which were induced by the HDAC inhibitors in cells refractory to lytic induction. Therefore, VPM selectively inhibits both viral and cellular gene expression. VPA and VPM represent a new class of antiviral agents. The mechanism by which VPA and VPM block EBV reactivation may be related to their anticonvulsant activity.

## IMPORTANCE

Epstein-Barr virus, (EBV), a human tumor virus, establishes a life-long latent infection. Reactivation of EBV into the lytic phase of its life cycle allows the virus to spread. Previously, we showed that EBV reactivation was blocked by valproic acid (VPA), an inhibitor of cellular histone deacetylases (HDACs). VPA alters the expression of thousands of cellular genes. In this study, we demonstrate that valpromide (VPM), an amide derivative of valproic acid that is not an HDAC inhibitor, prevented initiation of the EBV lytic cycle. VPA induced lytic reactivation of Kaposi’s sarcoma-associated herpesvirus (KSHV), but VPM did not. Unlike VPA, VPM did not activate cellular immediate-early gene expression. VPM is a new type of antiviral agent. VPM will be useful in probing the mechanism of EBV lytic reactivation and may have therapeutic application.

## INTRODUCTION

Epstein-Barr virus (EBV), a human gammaherpesvirus, causes infectious mononucleosis and other lymphoproliferative diseases. EBV is intimately associated with lymphomas and with carcinomas of the stomach and nasopharynx. Like all herpesviruses, EBV establishes a latent infection that is periodically reactivated into the productive lytic cycle. While the physiologic mechanisms by which the EBV lytic cycle is reactivated in immunocompetent people are not known, lytic reactivation can be triggered in cultured cells by various inducing agents, including the short-chain fatty acid butyrate (1). However, medium-chain fatty acids, including valproic acid (VPA), block reactivation of the EBV lytic cycle caused by inducing agents in Burkitt lymphoma cells (2).

VPA and butyrate are both histone deacetylase (HDAC) inhibitors. One potential mechanism of action to account for the differential effects of butyrate and VPA on EBV reactivation may lie in the specific modifications of chromatin that are produced by the two agents. However, a number of experiments have provided evidence that histone modification and EBV lytic reactivation do not always correlate. (i) VPA and butyrate both inhibit class I and IIa HDACs (3). (ii) Markers characteristic of open chromatin, namely, hyperacetylation of histone H3 at lysine 9 (K9) and K14 and dimethylation of H3 at K4, are globally induced in EBV-positive HH514-16 cells treated with VPA, yet VPA does not induce the viral lytic cycle in these cells (4). (iii) Markers of open chromatin, consisting of hyperacetylation of histones H3 (K9 and K14) and H4 (K5, K8, K12, and K16), and phosphorylation of serine 10 on histone H3 were induced by butyrate in Raji cells, yet the EBV lytic cycle was not activated. (iv) In HH514-16 cells treated with butyrate, hyperacetylation of histone H3 was detected both in the subpopulation of cells that entered the lytic cycle and in the cells that remained refractory to viral reactivation (5). (v) Investigations of histone modifications, specifically at promoters of viral lytic genes, revealed no differences in histone H3 hyperacetylation at the BZFL1 promoter in HH514-16 cells treated with butyrate or VPA. (vi) Furthermore, the HDAC inhibitory activity of a panel of structurally related short-chain fatty acids did not correlate with activation or blockage of EBV reactivation (2). Therefore, a mechanism other than HDAC inhibition must contribute to the blockade of EBV lytic reactivation by VPA.

Another possibility that could account for the differential effects of VPA versus butyrate on EBV reactivation is selective alteration of expression of cellular genes. Cellular gene expression is required before expression of viral transactivator genes (6). Butyrate may specifically activate expression of a gene required for EBV lytic activation, while VPA may activate a repressor. However, since butyrate and VPA are HDAC inhibitors, they each change the expression of thousands of genes. This makes the identification and characterization of specific genes required for either activating or repressing EBV lytic reactivation difficult. In fact, in cells treated with VPA or butyrate, the changes in cellular gene expression are largely overlapping (7).

**FIG 1:**
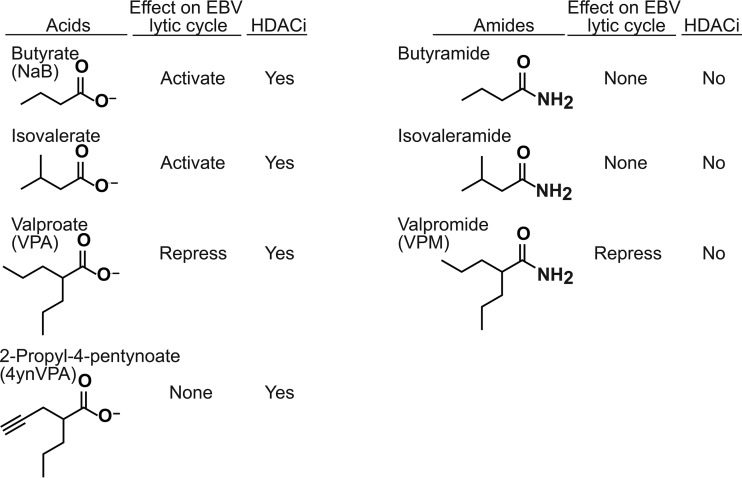
Structures of fatty acids and their amide derivatives studied for effects on the EBV lytic cycle. (Left column) Butyrate, isovalerate (3-methylbutyrate), VPA (2-propylpentanoate), and 4ynVPA (2-propyl-4-pentynoate). (Right column) Butyramide, isovaleramide, and VPM. HDACi, HDAC inhibitor.

In this report, we sought to determine whether the ability of VPA to block EBV reactivation is dependent on its property of inhibiting HDACs. Therefore, we examined valpromide (VPM), an analog in which the carboxylic acid of VPA is replaced with an amide (Fig. 1). This small change maintains the carbon chain length and branching structure of VPA, which we showed was important for the ability of VPA to block EBV reactivation (2). Importantly, however, VPM is not an HDAC inhibitor (8). We tested the effects of VPM and the amide derivatives of other fatty acids on EBV lytic reactivation. We found that VPM, like VPA, did not induce expression of the EBV lytic transactivator genes and blocked their expression induced by butyrate. Unlike VPA, VPM did not induce the lytic cycle of a related gammaherpesvirus, Kaposi sarcoma-associated herpesvirus (KSHV).

To initiate study of the mechanism(s) by which VPA and VPM block the EBV lytic cycle, we determined the effects of the two agents on expression of representative cellular genes that are either upregulated prior to EBV reactivation or enriched in refractory cells. We found that VPM specifically blocks the expression of two cellular immediate-early (IE) genes, FOS and EGR1, which are involved in EBV lytic reactivation. Thus, VPM is a novel selective inhibitor of EBV reactivation and cellular gene expression.

## RESULTS

### Valpromide, which is not an HDAC inhibitor, blocks EBV lytic reactivation in Burkitt lymphoma cells.

**FIG 2:**
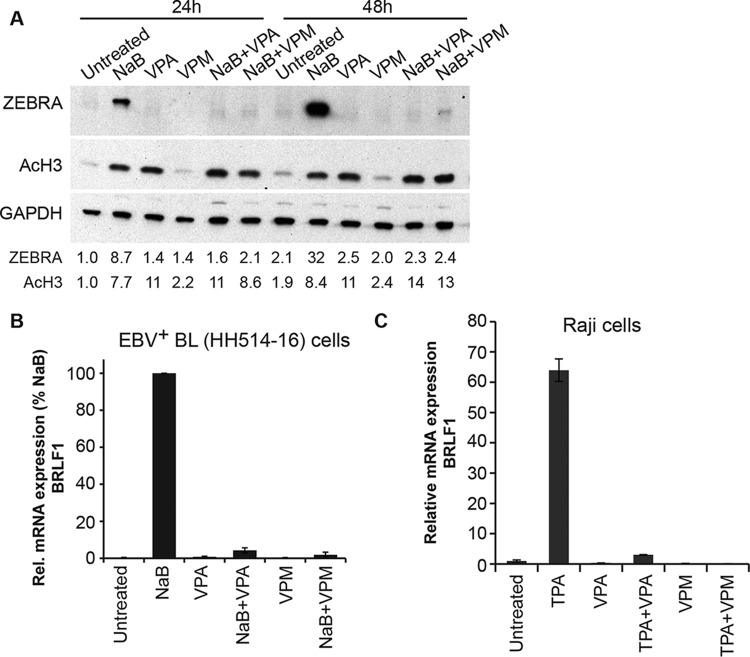
Valproic acid, an HDAC inhibitor, and valpromide, which is not an HDAC inhibitor, block reactivation of the EBV lytic cycle in two Burkitt lymphoma cells lines. (A) EBV in Burkitt lymphoma cells (HH514-16) were treated with VPA or VPM (10 mM) in the presence or absence of butyrate (NaB; 3 mM). After 24 or 48 h of treatment, lytic induction was measured by immunoblotting with an anti-Zebra antibody. HDAC inhibition was measured using an anti-acetyl H3 rabbit polyclonal antibody. (B) Protein levels were normalized to GAPDH levels and expressed relative to the amount in the untreated control cells at 24 h after treatment. EBV lytic induction was determined based on the relative expression of BRLF1 mRNA, measured by RT-qPCR. Data shown are the average results for biological triplicates expressed relative to stimulation by butyrate at 100%. Treatment with butyrate was the only condition significantly different (*P* < 0.05) from untreated cells. (C) EBV-infected Raji cells were treated with VPA or VPM (10 mM) in the absence or presence of TPA (20 ng/ml) for 18 h. Lytic induction was determined based on the relative expression of BRLF1, measured by RT-qPCR in triplicates of RNA extracted from untreated versus treated cells. Data represent results for biological duplicates.

VPA, an HDAC inhibitor, blocks lytic reactivation of EBV by all known inducing agents, including other HDAC inhibitors, in Burkitt lymphoma (BL) cells (7). To determine whether the blockade of EBV reactivation by VPA correlated with its HDAC inhibitory activity, we studied VPM (2-propyl-pentanamide), the carboximide derivative of VPA (Fig. 1). Pertinent to this study, VPM is not known to be an HDAC inhibitor (8). To compare the effects of VPA and VPM on the EBV lytic cycle, we treated EBV-positive Burkitt lymphoma cells (HH514-16) for 24 and 48 h with VPA or VPM (10 mM) in the presence or absence of butyrate (3 mM). VPM treatment did not alter cell viability, as determined by trypan blue staining. Cells treated with VPM did not exhibit increased levels of acetylated histone H3 (AcH3) (Fig. 2A), while those treated with butyrate and VPA, known HDAC inhibitors, manifest, as expected, increased acetylation of histone H3. Increased histone H3 acetylation was still observed in cells treated with butyrate combined with VPM, demonstrating that VPM does not block the HDAC-inhibitory property of butyrate.

We measured EBV reactivation in HH514-16 BL cells treated with butyrate, VPA, or VPM. The expression of the viral lytic transactivator protein Zebra was the marker of lytic reactivation. Zebra was detected in cells treated with butyrate, a known inducer of the EBV lytic cycle in this cell background, but not in cells treated with VPA or VPM (Fig. 2A). As shown previously, VPA blocked expression of Zebra by butyrate (7). VPM also inhibited Zebra expression induced by butyrate. In addition to blocking expression of Zebra protein, VPM blocked expression of BZLF1 mRNA (data not shown) and BRLF1 mRNA (Fig. 2B). VPM also blocked EBV reactivation induced by tetradecanoyl phorbol acetate (TPA), a protein kinase C agonist, in Raji cells, as indicated by inhibition of expression of viral immediate-early genes BZLF1 and BRLF1. This experiment showed that VPM inhibits the action of another inducing agent that works by a mechanism distinct from that of butyrate in a separate Burkitt lymphoma cell line (Fig. 2C).

### Effects of other derivatives of fatty acids on EBV lytic reactivation.

**FIG 3:**
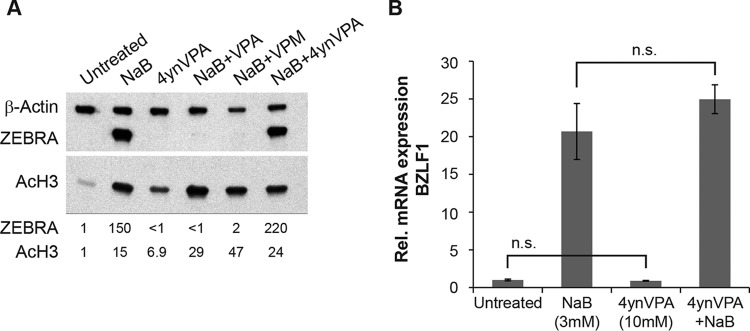
The VPA analog 4ynVPA, an HDAC inhibitor, does not activate or block EBV lytic reactivation. (A) HH514-16 EBV^+^ Burkitt lymphoma cells were treated with 4ynVPA (3 mM) in the presence or absence of butyrate (NaB; 3 mM) for 24 h. Lytic induction was measured by immunoblotting with anti-Zebra antibody. The assay for HDAC inhibition used an anti-acetyl H3 rabbit polyclonal antibody. Protein levels, normalized to β-actin levels, were expressed relative to the amount in the untreated control cells. (B) HH514-16 EBV^+^ Burkitt lymphoma cells were treated with 4ynVPA (10 mM) in the presence or absence of butyrate (3 mM) for 24 h. Lytic induction was determined based on the relative expression of BZLF1, measured by RT-qPCR, in triplicates of extracted RNA. Data are representative of results with biological duplicates. n.s., not statistically significant (*P*  > 0.05).

We investigated the effects on lytic reactivation of another derivative of VPA, 2-propyl-4-pentynoic acid (4ynVPA; ABS205; Abcam) (Fig. 1). 4ynVPA is a known HDAC inhibitor (9), like VPA. In HH514-16 Burkitt lymphoma cells treated with 4ynVPA, levels of AcH3 were increased, although not to the extent following treatment with butyrate and VPA (Fig. 3A). Like VPA, 4ynVPA (10 mM) did not induce expression of BZLF1 mRNA (Fig. 3B) or protein (Fig. 3A). However, unlike VPA, 4ynVPA did not block induction of BZFL1 by butyrate. These results provide additional evidence that inhibition of HDACs is not sufficient for either promoting or inhibiting EBV lytic reactivation.

**FIG 4:**
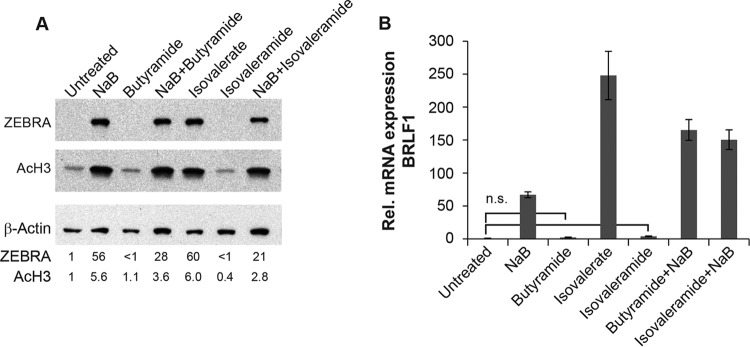
Amide derivatives of butyrate and isovalerate do not activate or block the EBV lytic cycle. HH514-16 EBV^+^ Burkitt lymphoma cells were treated with butyramide (10 mM) or isovaleramide (10 mM) in the presence or absence of butyrate (3 mM) for 24 h. (A) Lytic induction and HDAC inhibition were measured by immunoblotting, as described for Fig. 3. (B) Lytic induction was determined based on the relative expression of BRLF1, measured by RT-qPCR, in triplicate. Data are representative of results for biological triplicates. n.s., not statistically significant (*P*  > 0.05).

Because VPM, the amide derivative of VPA, was an effective inhibitor of EBV lytic reactivation, we investigated the properties of amide derivatives of butyrate and isovalerate, two fatty acid HDAC inhibitors that activate the EBV lytic cycle (2) (Fig. 4). Treatment of HH514-16 Burkitt lymphoma cells with the amide derivatives, butyramide and isovaleramide (Fig. 1), did not cause an increase in acetylated histone H3 (Fig. 4A). Neither butyramide nor isovaleramide induced expression of ZEBRA protein (Fig. 4A) or BRLF1 mRNA expression (Fig. 4B). Unlike VPM, neither butyramide nor isovaleramide blocked reactivation of the EBV lytic cycle, as measured by expression of Zebra protein or BRLF1 mRNA. This result showed that simply converting a fatty acid to the corresponding amide is not sufficient to create an EBV lytic cycle inhibitor. VPM is a novel inhibitor of EBV lytic reactivation. The structure of the carbon chain in VPM is crucial for its function in inhibiting EBV reactivation.

### VPM blocks butyrate-induced expression of the BZLF1 promoter.

**FIG 5:**
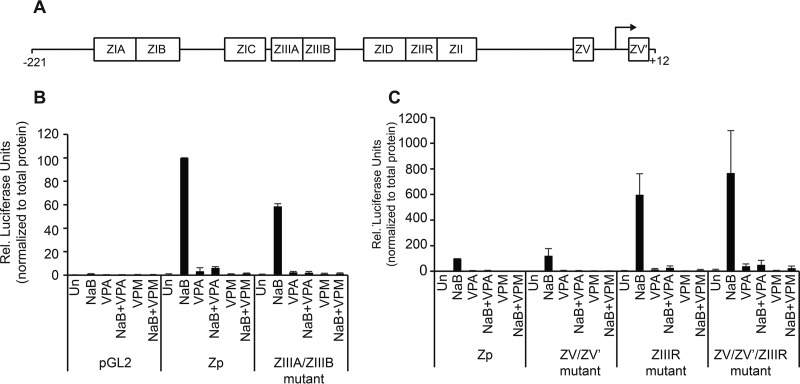
VPM blocks induction of expression of the BZLF1 promoter (Zp). (A) Schematic diagram of the EBV BZLF1 promoter (Zp) from positions −221 to +12 relative to the transcription start site, with known response elements labeled. (B and C) Effects of VPM (10 mM) compared to those with VPA (10 mM) or butyrate (3 mM) on the expression of luciferase regulated by Zp, either wild type or with inactivating mutations in the ZIIIA/ZIIIB, ZIIR, or ZV/ZV′ elements. The luciferase activities were normalized to total protein levels. The data are average results for at least three separate transfections. Un, untreated control.

VPA and VPM block accumulation of BZLF1 mRNA when BL cells are treated with an inducing agent (Fig. 2B). This activity could reflect effects of VPA and VPM on elongation or stability of BZLF1 mRNA or initiation of mRNA transcription. We showed previously that butyrate upregulates expression of a luciferase (luc) reporter driven by the BZLF1 promoter (Zp) and that VPA blocks Zp-luc expression by butyrate. Here, we show that VPM, like VPA, does not induce Zp-luc expression and blocks stimulation of this reporter by butyrate (Fig. 5). We tested a number of mutations in the Zp reporter in our attempt to map the locus of the inhibitory action of VPM. To test whether VPM blocks autoactivation of Zp, we created inactivating mutations in the ZIIIA/ZIIIB sites within Zp that are known to mediate autostimulation (10). The inhibitory effect of VPM was maintained on Zp reporters with inactivating mutations in the ZIIIA/ZIIIB autostimulatory sites (Fig. 5B). The inhibitory effect of VPM was also evident on Zp-luc reporters with inactivating mutations of previously mapped repressive elements, ZV/ZV′ and ZIIR (11, 12). Although the ZV/ZV′/ZIIR mutations in Zp-luc were accompanied by an 8-fold increase in Zp expression in response to butyrate (Fig. 5C), VPM still repressed the activity of butyrate. We conclude that neither VPA nor VPM blocks Zp expression via a repressive mechanism that is mediated by the ZV/ZV′ or ZIIR sites.

### VPA and VPM block EBV late protein expression in the presence of Zebra protein.

**FIG 6:**
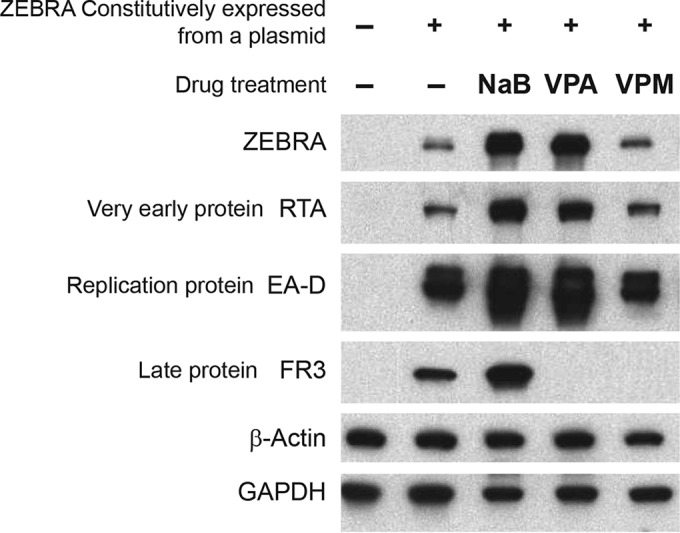
VPA and VPM block EBV late protein expression. HH514-16 EBV^+^ Burkitt lymphoma cells were transfected with a control vector or a plasmid expressing BZLF1 from the constitutive CMV promoter and then treated with butyrate (3 mM) or VPA or VPM (10 mM) for 48 h. Levels of EBV proteins Zebra, RTA, EA-D, and FR3 were detected by immunoblotting.

To determine whether or not VPA and VPM inhibit any other phase of the lytic cycle kinetically downstream of BZLF1 expression, we transfected HH514-16 BL cells with a plasmid that constitutively expressed BZLF1 and then treated the cells with butyrate, VPA, or VPM or left them untreated. Zebra protein was expressed from the plasmid in cells treated with VPM at a level similar to that in untreated cells (Fig. 6). Zebra expression was increased in the presence of the HDAC inhibitors butyrate or VPA, due to enhanced expression from the cytomegalovirus (CMV) IE promoter in the plasmid (13). The levels of expression of the viral transactivator RTA (BRLF1 gene) and early EA-D (BMRF1) proteins mirrored the expression pattern of Zebra. The levels of Rta and EA-D were enhanced in the presence of butyrate and VPA; their levels were similar in cells treated with VPM or untreated after transfection with BZLF1. These results showed that neither VPA nor VPM blocks the function of Zebra in activating early protein expression. However, late protein expression of small capsid protein FR3 (BFRF3) was inhibited by VPA and VPM, but not by butyrate. In these cells in which FR3 protein was not detected, lytic EBV DNA replication was detected by quantitative PCR (qPCR). Therefore, VPA and VPM block the EBV lytic cycle at two steps: very early gene expression (BZLF1) and late protein (FR3) expression.

### Comparison of effects of VPA and VPM on cellular gene expression.

**FIG 7:**
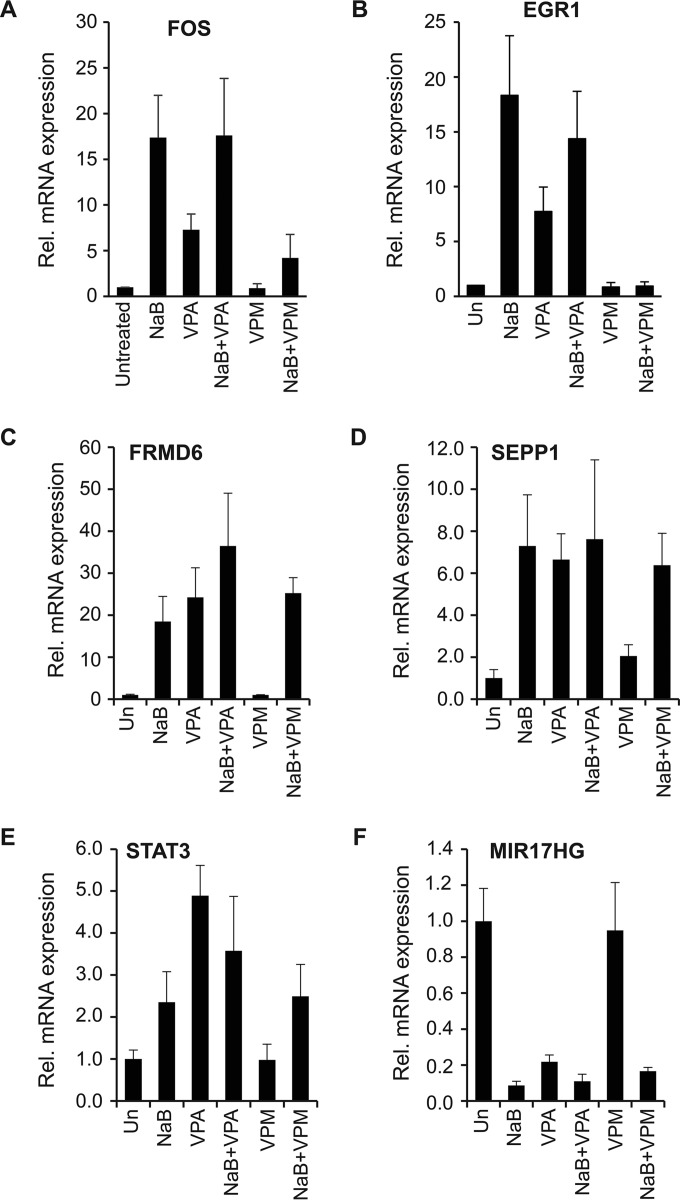
Increased expression of cellular immediate-early genes, EGR1 and FOS, induced by butyrate is blocked by VPM. HH514-16 EBV^+^ Burkitt lymphoma cells were treated with VPA or VPM (10 mM) in the presence or absence of butyrate (3 mM) for 6 h. Relative mRNA expression was measured by RT-qPCR.

In previous work, we showed that expression of cellular immediate-early genes, including FOS and EGR1, is temporally upstream of expression of the viral BZLF1 and BRLF1 genes in two BL cell lines, Akata and HH514-16 cells, treated with lytic cycle-inducing agents appropriate for each cell line, namely, anti-Ig for Akata and butyrate or trichostatin A for HH514-16 (7, 14). We compared the effects of VPA and VPM on expression of cellular immediate-early genes EGR1 and FOS in HH514-16 cells. VPA induced expression of EGR1 and FOS, though to a lesser extent than butyrate, in HH514-16 cells treated for 6 h. VPA did not inhibit the induction of these two cellular genes by butyrate. In contrast, VPM did not promote expression of EGR1 or FOS and VPM inhibited their induction by butyrate (Fig. 7A and B). Since VPM is not an HDAC inhibitor, its failure to promote expression of EGR1 and FOS was not unexpected. However, the inhibitory effect of VPM on EGR1 and FOS expression showed that VPM and VPA differ in their modulating effects on cellular gene expression.

We expanded the comparison of VPA and VPM by examining their effects on four cellular genes whose expression is enriched in HH514-16 BL cells that are refractory to lytic induction (5). The two HDAC inhibitors, butyrate and VPA, induced expression of FRMD6, SEPP1, and STAT3 at 6 h. VPM did not enhance the expression of these genes compared to untreated control cells (Fig. 7C to E). In addition, VPM did not inhibit induction of FRMD6, SEPP1, or STAT3 by butyrate. The two HDAC inhibitors butyrate and VPA decreased expression of the MIR17HG locus; VPM did not alter expression of this locus (Fig. 7F). Expression of MIR17HG was still decreased by butyrate, even in the presence of VPM. The effects of the HDAC inhibitors butyrate and VPA on the expression of FRMD6, SEPP1, STAT3, and MIRF17HG differed from the effects of the non-HDAC inhibitor VPM. Since VPM did not impact the alterations in gene expression induced by butyrate, we conclude that VPM did not block EBV lytic reactivation by facilitating expression of these four genes that are characteristic of the refractory state. While VPM inhibited expression of two immediate-early cellular genes but did not alter expression of four genes upregulated in refractory cells, VPM is not a global inhibitor but is a selective inhibitor of cellular gene expression.

### Valpromide does not induce or block the KSHV lytic cycle.

**FIG 8:**
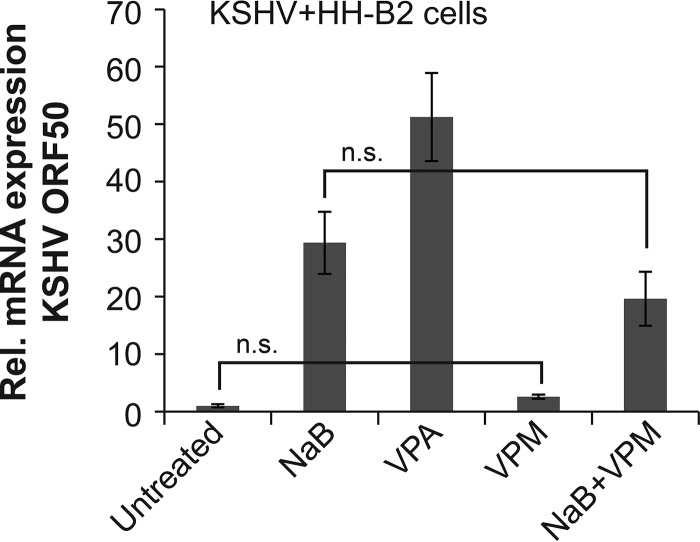
VPM does not reactivate the lytic cycle of KSHV. KSHV in primary effusion lymphoma cells (HH-B2) was treated with butyrate (NaB; 3 mM), VPA (10 mM), or VPM (10 mM) for 12 h. The relative expression of KSHV ORF50 was measured by RT-qPCR in triplicate samples of RNA. Data are representative of biological triplicate experiments. n.s., not statistically significant (*P*  > 0.05).

Although VPA blocks lytic reactivation of EBV in Burkitt lymphoma cells, VPA activates the lytic cycle of KSHV, another human gammaherpesvirus in primary effusion lymphoma (PEL) cells (15). Our earlier work showed that, unlike EBV, KSHV lytic reactivation correlates with HDAC inhibition by short-chain fatty acids (2). We postulated that VPM, which is not an HDAC inhibitor, would not activate KSHV. In KSHV-positive HH-B2 PEL cells treated with VPM, expression of the viral transactivator ORF50 gene was not induced (Fig. 8). In addition, the ORF50 gene was still expressed in cells treated with VPM plus butyrate. Therefore, VPM neither activates nor blocks KSHV lytic reactivation. VPM is a not a general inhibitor of viral lytic reactivation.

## DISCUSSION

### Structure-function relationship among inhibitors of EBV lytic reactivation that are antiepileptic agents.

VPA is widely used to treat epilepsy, mood disorders, and migraine headaches. Despite its widespread use, VPA can cause serious side effects, including liver toxicity and teratogenicity. To identify antiepileptic drugs with reduced side effects, derivatives of VPA have been synthesized. By utilizing these derivatives of VPA, a previously identified inhibitor of EBV reactivation, we are able to provide additional insights into structural features of fatty acids that are important for inhibition of the lytic cycle of EBV. 4ynVPA (Fig. 1), containing a triple bond in one of the carbon side chains of VPA, retains some anticonvulsant activity but is less effective than VPA (16). The *S* isomer of 4ynVPA, like VPA, is an HDAC inhibitor (9) but is more teratogenic than VPA (17). Teratogenicity correlates with HDAC inhibition (18). In our studies, a racemic mixture of 4ynVPA behaved as an HDAC inhibitor but neither activated, nor blocked, EBV lytic reactivation (Fig. 3). This result confirmed our previous conclusions that neither the stimulatory nor inhibitory effects of fatty acids on EBV reactivation correlate with HDAC inhibition (2). The anticonvulsant properties of VPA are maintained in analogs that are not HDAC inhibitors (18, 19). Converting the carboxylic acid in VPA to an amide creates VPM (Fig. 1). VPM, like VPA, is an antiepileptic drug. However, VPM is neither a teratogen (20) nor an HDAC inhibitor (21). Here, we report that VPM is a novel inhibitor of EBV reactivation (Fig. 2).

We hypothesize that the effects of VPA and VPM in blocking EBV reactivation are related to their properties as anticonvulsants. The precise mechanism of action of VPA as an antiepileptic drug is not known. A number of activities have been postulated, such as effects on ion channels, GABAergic and glutamatergic neurotransmitter activities, and cell signaling kinases, including inositol-dependent signaling (22).

### VPM blocks upregulation of cellular immediate-early genes by butyrate.

The EBV lytic cycle can be triggered by many different molecules with disparate effects on the host cell, including protein kinase C agonists, cross-linking of the B-cell receptor by anti-immunoglobulin, the DNA demethyltransferase inhibitor azacytidine, and many different classes of HDAC inhibitors, including butyrate, trichostatin A, and suberoylanilide hydroxamic acid (7). Cells react to these stimuli by altering expression of cellular IE genes that characteristically respond rapidly to extracellular signals such as hormones or stress. Activation of expression of the EBV transactivator genes, BZLF1 and BRLF, occurs after expression of cellular immediate-early genes, including EGR1 and FOS (14). Evidence suggests that EGR1, FOS, and other cellular IE genes play an essential role in EBV lytic reactivation. EGR1 activates the promoter of the EBV BRLF1 gene (23). Transfection of Akata cells with plasmids expressing EGR1 and other cellular immediate-early genes, namely, EGR2, NR4A, and NR4A3, results in increased EBV BZLF1 expression (14). Knockdown of EGR1 reduces spontaneous expression of BZLF1 and BRLF1 (24). Since EGR1 expression is enhanced by Zebra, there may be a positive feedback loop stimulating EGR1 action on the BZLF1 and BRLF1 promoters. The BZLF1 promoter (Zp) is also stimulated by overexpression of FOS/JUN, an effect that is enhanced by treatment with transforming growth factor-β (TGF-β) and overexpression of Smad3/Smad4 (25). Stimulation of Zp was independent of Zebra protein, since Zp reporter activation by FOS/JUN occurred in both EBV-positive and EBV-negative cell lines.

The mechanisms by which VPM and VPA block EBV lytic reactivation may involve altering expression of one or more cellular immediate-early genes that influence expression of the EBV transactivator genes. VPM did not enhance the expression of EGR1 or FOS. In fact, VPM blocked upregulation of EGR1 and FOS by butyrate (Fig. 7). It is plausible that blockade of expression of cellular IE genes contributes to mechanisms by which VPM inhibits the expression of EBV transactivator genes. However, VPA stimulates the expression of EGR1 and FOS; therefore, the mechanisms by which VPM and VPA regulate cellular gene expression differ. If there are common ways in which VPA and VPM block EBV lytic reactivation via regulation of cellular immediate-early genes, genes other than EGR1 and FOS must play a role.

### Potential therapeutic use of VPA and VPM in medicine.

Inhibitors of the EBV lytic cycle are candidates for treatment of diseases in which there is a high viral load of EBV associated with increased levels of lytic replication. Currently utilized antiherpesvirus drugs target viral DNA replication. Since VPM and VPA target two additional steps in the viral lytic pathway, namely, reactivation from latency and expression of viral late proteins (Fig. 2 and 6), VPA and VPM might prove useful for therapy of diseases with increased lytic replication. Primary EBV infection during adolescence causes infectious mononucleosis, a disease characterized by high levels of lytic replication in the oropharynx and a high viral load in the blood. Oral hairy leukoplakia results from lytic replication of EBV in the tongue (26). The lytic cycle of EBV also plays a critical role in oncogenesis. Increased viral load precedes the onset of nasopharyngeal carcinoma, Burkitt lymphoma, and Hodgkin lymphoma by months to years (27–29). Suppression of the immune system during AIDS or following organ transplantation results in reactivation of EBV, high viral loads, and increased risk of cancer. Since the inhibitory effects of VPA and VPM described here occur in lymphoid cells, drugs of this class may find use in treatment of patients with high viral loads secondary to immunosuppression with a risk of developing lymphoid malignancies.

### Reactivation or inhibition of other viruses.

An important issue to consider in the use of VPA or VPM in patients with EBV-associated diseases is their potential to reactivate coexisting latent viral infections, particularly with other herpesviruses, which are common and establish a lifelong latent infection. The human herpesvirus most closely related to EBV is KSHV. The lytic cycle of KSHV is induced by VPA (15). However, since VPM does not induce lytic KSHV in primary effusion lymphoma cells (Fig. 8), VPM might be preferable to VPA for treatment of patients coinfected with EBV and KSHV. Among other herpesviruses, pretreatment of cells with VPA results in increased expression of CMV lytic antigens and stimulates CMV replication (30–32). Treatment with VPA increases expression of the CMV IE promoter reporter (33). Our results also show increased expression of Zebra from the CMV IE promoter by VPA, and also by butyrate (Fig. 6). However, the level of expression of Zebra from the plasmid driven by the CMV IE promoter in cells treated with VPM was the same as in untreated cells (Fig. 6). Thus, a potential advantage of the use of VPM is that it does not reactivate KSHV or stimulate the CMV IE promoter. Further work is needed to assess whether VPM has an inhibitory effect on the replication of CMV or other members of the herpesvirus family.

In conclusion, the novel observations reported herein are useful to generate hypotheses for future studies to unravel the mechanistic details of the pathways of EBV lytic reactivation. VPM is likely to specifically impact cellular gene expression or other aspects of cellular physiology that are crucial for viral reactivation. Our discovery that VPM is an inhibitor of at least two steps in EBV lytic reactivation, without causing HDAC inhibition, allows investigation of changes in cellular gene expression or other aspects of cellular physiology that control or modify different temporal stages of the EB viral life cycle. Targeting EBV lytic reactivation with drugs such as VPM may prove to have prophylactic or therapeutic potential for EBV-associated malignancies in which lytic reactivation precedes or accompanies development of the cancer.

## MATERIALS AND METHODS

### Cell lines.

The HH514-16 Burkitt lymphoma cell line is a subclone of the EBV-infected P3J-HR-1 cell line (34). Raji is a Burkitt lymphoma-derived cell line (35). HH-B2 is a KSHV-infected primary effusion lymphoma cell line (36). Cells were cultured in RPMI 1640 containing 8% fetal bovine serum, penicillin (50 U/ml), streptomycin (50 U/ml), and amphotericin B (1 µg/ml).

### Chemical treatment and transfection of cell lines.

Cells were subcultured to 3 × 10^5^ cells/ml 48 h prior to each experiment. Cells at 1 × 10^6^/ml were treated with chemical stimuli for the durations noted in the figure legends. Sodium butyrate (NaB) and sodium valproate (VPA) (Sigma) were prepared as 1 M solutions in water. VPM (Alfa Aesar), TPA (Calbiochem), 4ynVPA (ABS205; Tocris/Santa Cruz Biotechnology), and butyramide and isovaleramide (Sigma) stocks were made in dimethyl sulfoxide. The chemical agents were added at the following concentrations: butyrate (3 mM), VPA or VPM (10 mM), TPA (20 ng/ml). Cell viability was measured by counting cells that excluded staining with trypan blue. In all drug treatment experiments, the cell viability was ≥90%. Cells (5 × 10^6^) were transfected using nucleofection (Lonza) with either 2 µg of an empty vector with a CMV promoter or 2 µg of a plasmid encoding Zebra. In some experiments (Fig. 6), chemical agents were added 1 h after transfection.

### Western blot analysis.

Cells that were untreated or treated with chemical stimuli were harvested at the times indicated in the figure legends. Total cell extracts were electrophoresed in 12% TGX SDS-polyacrylamide gels (Bio-Rad) and were transferred to nitrocellulose membranes. Rabbit polyclonal antibodies were used to detect histone H3 acetylated at lysine 9 and lysine 14 (catalog number 06-599; EMD Millipore) and EBV BFRF3. Mouse monoclonal antibodies were used to detect Zebra (BZ1) (37), EA-D, glyceraldehyde-3-phosphate dehydrogenase (GAPDH; ab8245; Abcam), and β-actin (A5316; Sigma). Protein levels were determined by densitometry.

### Reverse transcription-quantitative PCR.

Total RNA was isolated using an RNeasy kit with on-column DNase digestion (Qiagen). Relative transcript levels were determined using gene-specific primers of the iScript SYBR green reverse transcription-quantitative PCR (RT-qPCR) kit (Bio-Rad). Primers for the EBV and cellular mRNAs studied have been described previously (6, 7). Relative expression levels were calculated using the ΔΔ*C_q_* (quantification cycle) method and were normalized to 18S RNA. RNA samples were assayed in triplicate. Statistical significance was calculated by using the paired *t* test.

### Luciferase reporter assays.

The fragment of the BZLF1 promoter (Zp) from positions −221 to +12 relative to the transcription start site was PCR amplified from the EBV genome of HH514-16 cells and subcloned into the PGL2-Basic vector to create Zp-luc. Mutations in Zp reported to disrupt the activity of the ZIIIA/ZIIIB, ZIIR, and ZV/ZV′ response elements (10–12) were generated as previously described (2). HH514-16 cells were transfected by nucleofection with 1 µg PGL2-Basic or a Zp reporter construct and treated with drugs at 1 h posttransfection. Cells were harvested at 48 h posttransfection and lysed in cell culture lysis reagent (Promega). Luciferase assays were performed using a luciferase assay system (Promega). Relative luciferase units for each sample were normalized to the amount of total protein determined with a bicinchoninic acid (BCA) assay (Pierce) with cell lysates pretreated with 2 volumes of iodoacetamide (100 mM) at 37°C for 15 min.
